# Improvement in the breakdown endurance of high-*κ* dielectric by utilizing stacking technology and adding sufficient interfacial layer

**DOI:** 10.1186/1556-276X-9-464

**Published:** 2014-09-03

**Authors:** Chin-Sheng Pang, Jenn-Gwo Hwu

**Affiliations:** 1 Department of Electrical Engineering and the Graduate Institute of Electronics Engineering, National Taiwan University, Taipei 10617, Taiwan

**Keywords:** MOS, HfO_2_/SiO_2_, Stacking structure, Time-zero dielectric breakdown, Interfacial layer, Interface trap density, Nitric acid oxidation

## Abstract

Improvement in the time-zero dielectric breakdown (TZDB) endurance of metal-oxide-semiconductor (MOS) capacitor with stacking structure of Al/HfO_2_/SiO_2_/Si is demonstrated in this work. The misalignment of the conduction paths between two stacking layers is believed to be effective to increase the breakdown field of the devices. Meanwhile, the resistance of the dielectric after breakdown for device with stacking structure would be less than that of without stacking structure due to a higher breakdown field and larger breakdown power. In addition, the role of interfacial layer (IL) in the control of the interface trap density (*D*_it_) and device reliability is also analyzed. Device with a thicker IL introduces a higher breakdown field and also a lower *D*_it_. High-resolution transmission electron microscopy (HRTEM) of the samples with different IL thicknesses is provided to confirm that IL is needed for good interfacial property.

## Background

As the shrinking of devices continues, conventional metal-oxide-semiconductor field-effect transistor (MOSFET) will reach the dimension limitation because of excessive gate leakage current, which would result in an increase in static power consumption and error read in logic device [1]. In addition, since the distance needed to obtain full bandgap SiO_2_ at each interface is about 3.5 ~ 4 Å, thickness of 8 Å is required for a perfect dielectric [2,3]. Under the situation, it is expected that the physical limited thickness for SiO_2_ is about 8 Å. Moreover, because the dimension of device decreases more rapidly in comparison with operating voltage, electric field applied upon the gate dielectric would increase more quickly. Therefore, severe phonon scattering and downgraded channel mobility would happen since channel carriers would be attracted towards the dielectric interface. The study of Timp et al. [4] revealed that the drive current of device would decrease while SiO_2_ thickness is less than 13 Å.

An obvious solution to the above problem is achieved by applying material with higher permittivity (high-*κ*) than SiO_2_, since it could not only suppress the gate leakage current but also maintain the same oxide capacitance. Numerous studies of high-*κ* materials such as HfO_2_, HfSiON, Al_2_O_3_, ZrO_2_, Ta_2_O_5_, TiO_2_, Y_2_O_3_, SrTiO_3_ (STO), and BaSrTiO_3_ (BST) were proposed as potential candidates for replacing SiO_2_. However, materials with merely medium permittivity of *κ* < 10 [5,6] would not achieve significant advantage over SiO_2_ when the dielectric becomes thinner. In addition, high-*κ* materials such as Ta_2_O_5_ and TiO_2_[7] would result in thermal instability while contact directly to Si. While for the STO and BST, some reports revealed that the high dielectric constant would result in fringing field-induced barrier-lowering effect and would cause a short channel effect [8].

HfO_2_ is a very promising high-*κ* material and owns several advantages in the formation of gate dielectric layer. It owns high dielectric constant (*κ* ~ 20), relatively large bandgap (5.7 eV) [9], and high heat of formation (271 kcal/mol) [10]. Great numbers of research in the fabrication of high-*κ* dielectric films had been reported [9-16]. Atomic layer deposition (ALD) is generally reported as a good method to form HfO_2_. However, there still exist some technique concerns about the degradation of metal-oxide-semiconductor (MOS) device reliability [17,18].

The method of nitric acid oxidation (NAO) was adopted in this work [19]. Noticeably, this method is not only cost-effective but could also be carried out in a low temperature (below 323 K in the whole process). The process is proceeded by the reaction of Hf with atomic oxygen which is produced by the decomposition of HNO_3_ according to the reaction 2HNO_3_ → 2NO + H_2_O + 3O. The high-*κ* HfO_2_ dielectric layer can be formed by NAO towards sputtered Hf metal layer due to the high reactivity of atomic oxygen. The method of NAO is also available in forming Al_2_O_3_ from Al metal [20]. Some research focused on the enhancement of illumination and temperature sensitivity by using NAO process to form HfO_2_ on interfacial layer (IL) [21,22]. Furthermore, since NAO is carried out at room temperature, multi-stacking structures could be achieved without the consideration of thermal budget, and each stacking layer could also be fully oxidized in order to reach optimal quality of dielectric structure. Several studies on the trapping characteristics of stacking structure Al_2_O_3_ and HfO_2_ had been proposed [23,24]. The research of tunneling current characteristics in dark and illumination was also explored on stacking structure [21]. It is believed that the process control of stacking technology for devices with better performance and reliability is still of interest.

The importance of IL is also examined in this work. Numerous reports demonstrated that an intentionally grown ultrathin oxide IL is indeed necessary to maintain stability between HfO_2_ and Si [25,26]. HfO_2_ film is believed to have poor interface property with Si which may be caused by the undercoordinated hafnium atom, so the electrical properties of dielectrics would not be optimized [27-29]. Additionally, nonuniformity and poor morphology for HfO_2_ film growing on hydrofluoric (HF)-last Si were found according to high-resolution transmission electron microscopy (HRTEM) and MEIS analyses. Since it is difficult to form a high-*κ* dielectric that having perfect interface with Si in comparison with SiO_2_, the use of SiO_2_ as IL is crucial and needed [30,31]. Moreover, the IL could not only help to reduce the thermodynamic instability between high-*κ* materials and Si, but it could also accommodate the difference in lattice constants between Si and another material.

In this work, we first manufactured two batches of MOS capacitors with the first batch having one-time forming HfO_2_ with SiO_2_ as gate dielectric layers and the second batch of HfO_2_ stacking layer with SiO_2_ as gate dielectric layers. The time-zero dielectric breakdown (TZDB) tests are investigated, and the current–voltage (I-V) characteristics are discussed. It is found that stacking structure owns a higher breakdown field, which would lead to lower resistance after breakdown. Then, in order to corroborate the results, samples with different IL thicknesses are manufactured and investigated. The stacking structures still own a higher breakdown field. Nevertheless, with the decreasing thickness of IL, higher density of interfacial states and lower breakdown field are observed. The mechanism for the observation is proposed, and HRTEM is given in this work.

## Methods

Two different MOS capacitors studied in the first experiment denoted by SH/O and H/O (S stands for stacking structure, H stands for HfO_2_, and O stands for SiO_2_) were manufactured on the substrate of p-type (100) Si wafer with a resistivity of 1 ~ 10 Ω cm. The wafers were undergone the process of standard Radio Corporation of America (RCA) cleaning in order to remove impurities. Then, SiO_2_ as ultrathin IL was grown onto the wafers using the technique of anodization (ANO) after removing native oxides by HF. The oxidation method of ANO could be carried out in room temperature and could provide a promising option for the preparation of low-temperature IL [32,33]. It was reported that the anodic oxide grown in room temperature has few pinholes and owns a good dielectric quality [34,35]. The samples after anodization were followed by 950°C annealing in N_2_ for 15 s. Then, sample H/O was undergone the deposition of Hf onto a wafer by sputtering with the power of 60 W for 210 s, followed by NAO process to form HfO_2_ dielectric. Then, postoxidation annealing (POA) was carried out in a furnace at 380°C for 10 min in order to improve the quality of dielectric layer. The combined procedures from the deposition of Hf to the following annealing are defined as one cycle. Under the circumstance, the sample SH/O would undergo the sputtering time of 90 s as the first cycle and that of 60 s as other two cycles. Then, 250-nm aluminum metal was evaporated onto the top of all samples. The process of photolithography was carried out to pattern the devices with square area of 2.25 × 10^4^ μm^2^. Finally, the back contact was formed by the evaporation of 250-nm aluminum.

**Figure 1:**
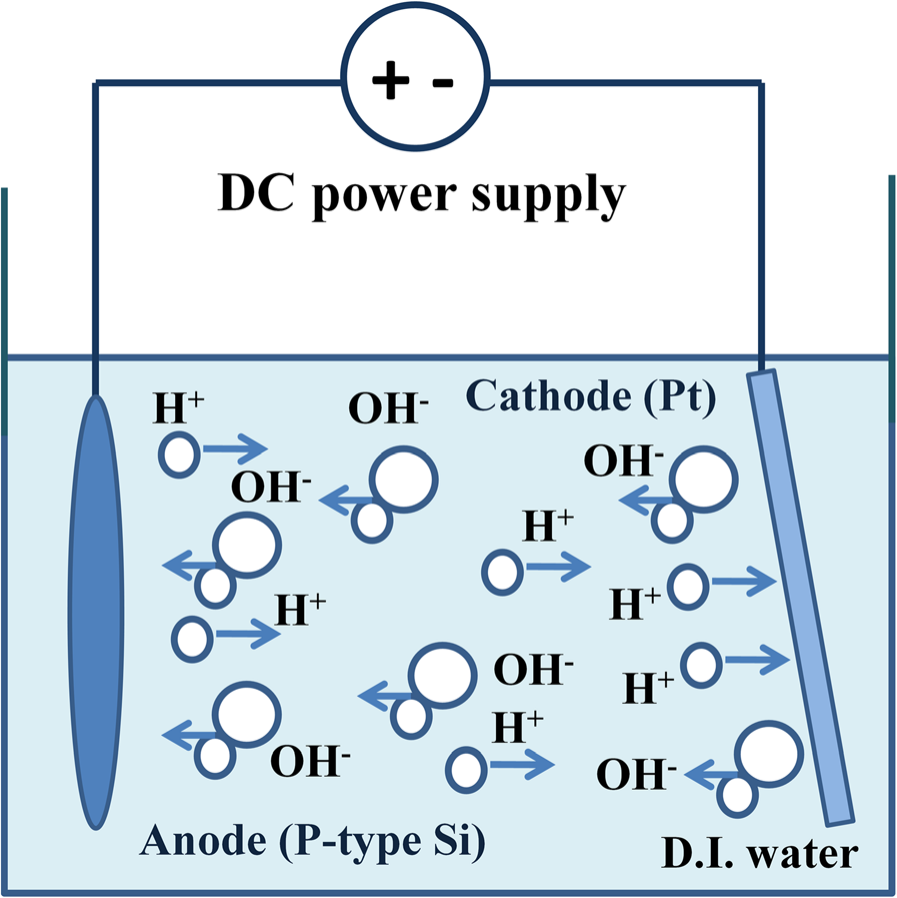
**The illustration of tilted platinum while using ANO process.** Silicon is connected to anode, while Pt is connected to cathode. During ANO, OH^-^ may be attracted to silicon, leading to the formation of SiO_2_.

In order to corroborate our investigation, another two different MOS capacitors with various IL thicknesses denoted by SH/O_x_ and H/O_x_ were manufactured. O_x_ represents the SiO_2_ that was formed with various thicknesses from ANO process. There are two main differences of the experiments for SH/O_x_ and H/O_x_ in comparison with SH/O and H/O. First, the platinum was tilted while using the ANO in order to form IL with different thicknesses, as shown in Figure 1. Second, H/O_x_ was undergone one cycle of Hf sputtering with 150 s instead of 210 s for H/O while SH/O_x_ was carried out Hf sputtering of 90/30/30 s separately instead of 90/60/60 s for SH/O.

## Results and discussion

### TZDB characteristics between one-time forming HfO_2_ and stacking structure

**Figure 2:**
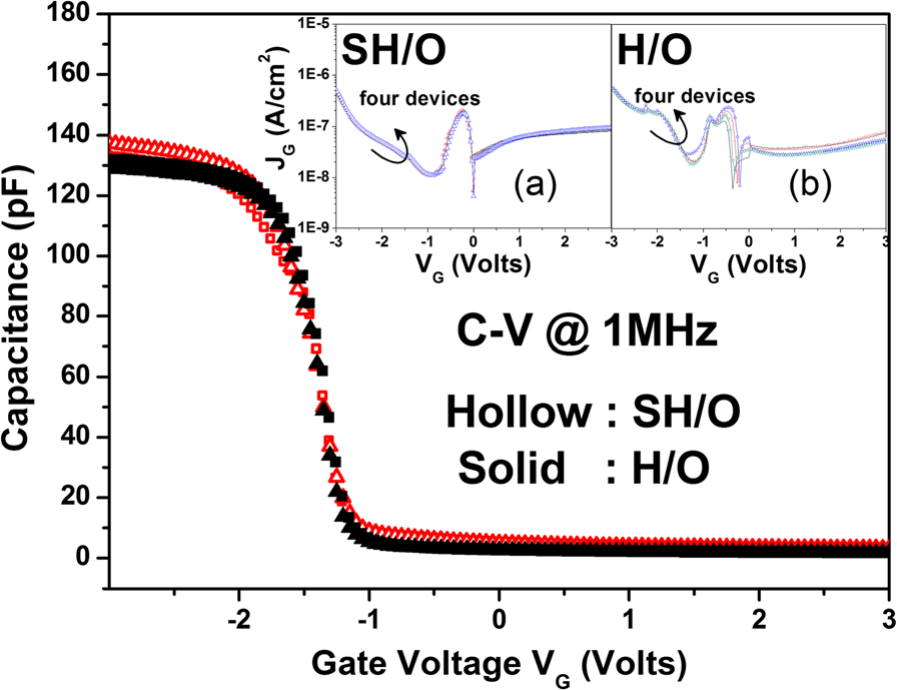
**C-V characteristics of stacking HfO**_**2**_**/SiO**_**2 **_**(SH/O) and single HfO**_**2**_**/SiO**_**2 **_**(H/O).** The I-V measurements for samples SH/O and H/O are shown in the insets **(a)** and **(b)**, respectively.

**Figure 3:**
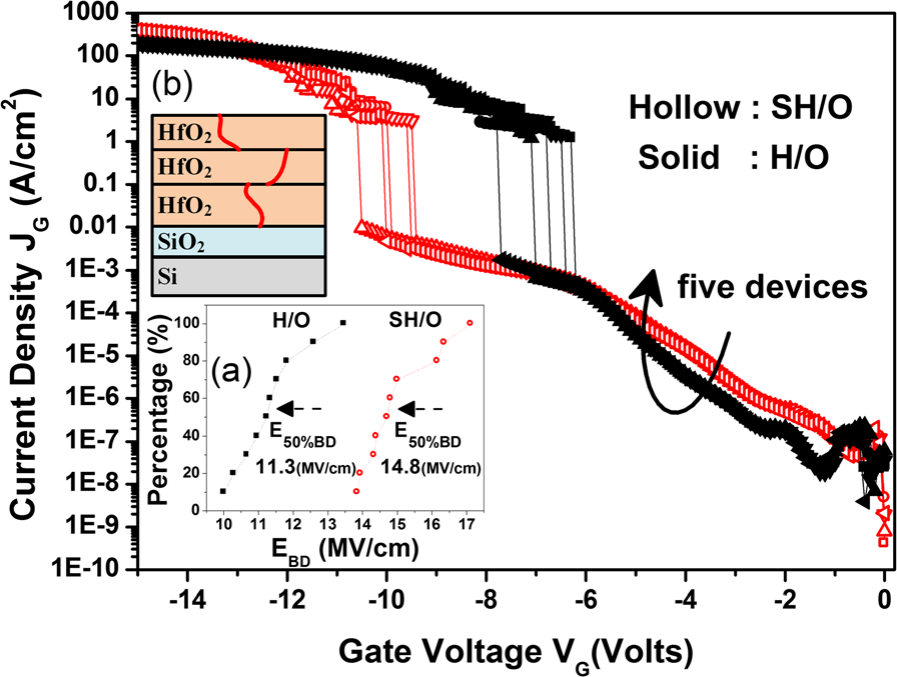
**I-V characteristics from *V***_***G***_ **= 0 to -15 V for SH/O and H/O. (a)** The cumulative data of *E*_BD_ for above samples. **(b)** The schematic illustration of possible leakage path in the stacking structure.

We first take the capacitance-voltage (C-V) and I-V measurements of H/O and SH/O. C-V measurements with gate voltage (*V*_*G*_) from -3 to 3 V are shown in Figure 2. Effective oxide thickness (EOT) of both samples is calculated as 52 Ǻ. The I-V curves of both devices are shown in the insets. In the following work, the TZDB characteristics are investigated. *V*_*G*_ is swept from 0 to -15 V in recording the leakage current density. It is observed that SH/O shows a higher breakdown voltage than the one without stacking structure as presented in Figure 3. Figure 3a presents the median breakdown field (*E*_50%BD_) of 14.8 (MV/cm) for SH/O, while merely 11.3 (MV/cm) for H/O. It is believed that the grain boundaries (GBs) exist in dielectric layer are responsible for current conduction [36]. It is supposed that the stacking structure would result in the misalignment of GBs between separate dielectric layers. With the discontinuous paths for current leakage as schematically illustrated in Figure 3b, the higher breakdown field (*E*_BD_) would be expected for stacking structure.

### Characteristics after dielectric breakdown

**Figure 4:**
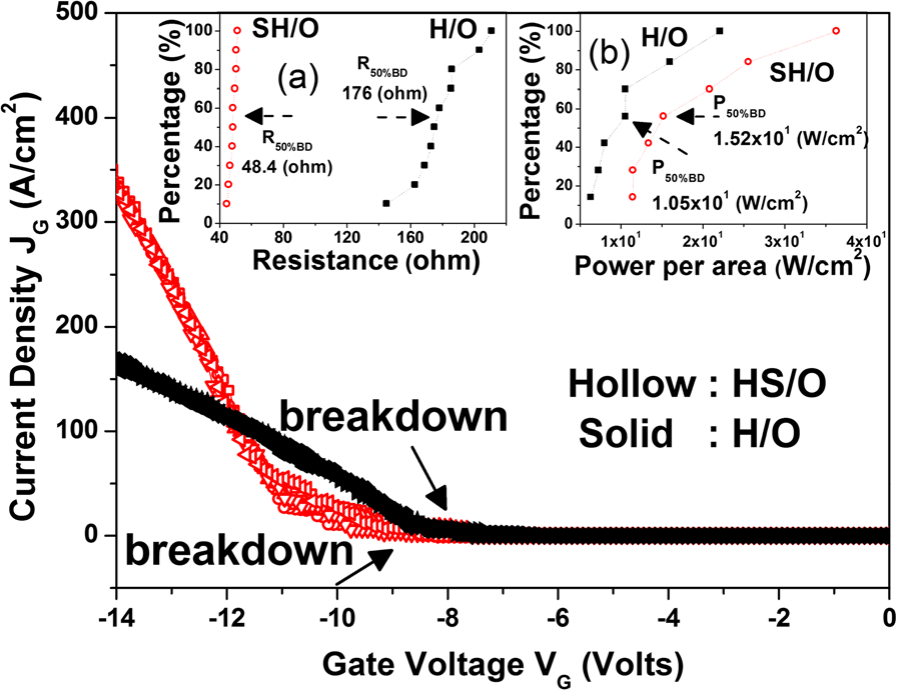
**I-V characteristics from *V***_***G***_ **= 0 to -15 V in linear scale for SH/O and H/O.** The cumulative data of resistance after breakdown and power per unit area at the initiation of breakdown for samples are shown in **(a)** and **(b)**, respectively.

The I-V characteristics after breakdown of these two samples are shown in Figure 4. Resistance after breakdown is defined as

(1) $$R=\frac{V_{1}‒V_{2}}{I_{1}‒I_{2}}\left(\mathrm{ohm}\right)$$

where *V* and *I* represent gate voltage and current. The cumulative data of *R* (absolute value) after breakdown are shown in Figure 4a. *R* is extracted with *V*_1_ and *V*_2_ of -13 and -12 V and the corresponding *I*_1_ and *I*_2_, respectively. It indicates that sample H/O shows higher *R* value than SH/O after breakdown. In the case, due to the finding that stacking structures have higher *E*_BD_, the power per unit area in the initiation of breakdown would be larger for stacking structures. The power per unit area of breakdown could be defined as

(2) $$P{'}_{\mathrm{BD}}=\mathrm{JV}\left(W/{\mathrm{cm}}^{2}\right)$$

where *J* and *V* are current density and corresponding gate voltage at the initiation of breakdown. The cumulative data of P'_BD_ are presented in Figure 4b. It indicates that the stacking structures would suffer from higher breakdown power per unit area than that of without stacking. It would lead to more serious damage in dielectric and result in lower resistance after breakdown.

### The important role of IL in reliability

**Figure 5:**
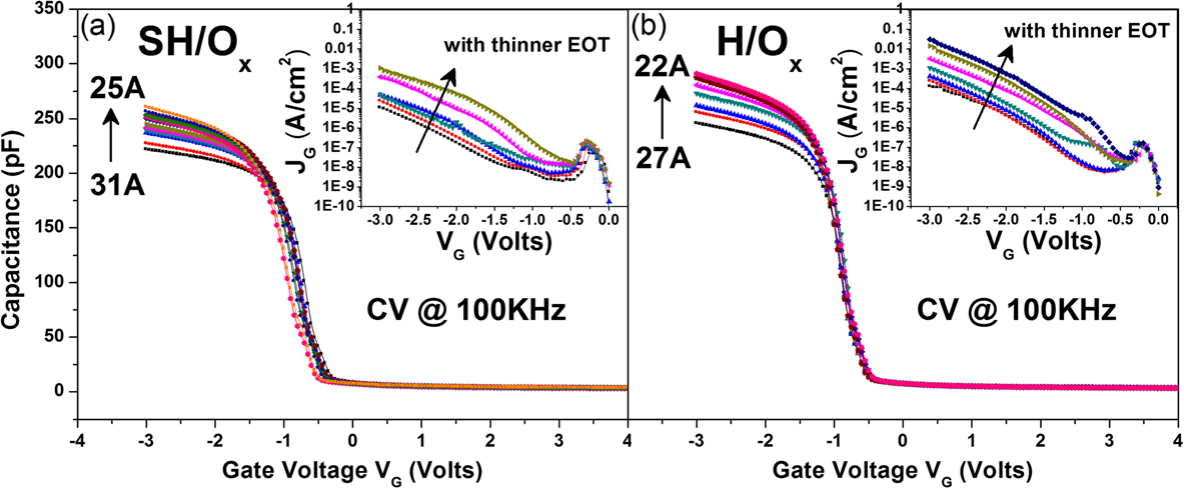
**C-V characteristics for samples with different EOTs due to different IL thicknesses. (a)** C-V curves for SH/O_x_ with EOT ranging from 25 to 31 Å. The I-V curves with different EOTs are shown in the inset. **(b)** C-V curves for H/O_x_ with EOT ranging from 22 to 27 Å. The I-V curves with different EOTs are shown in the inset.

**Figure 6:**
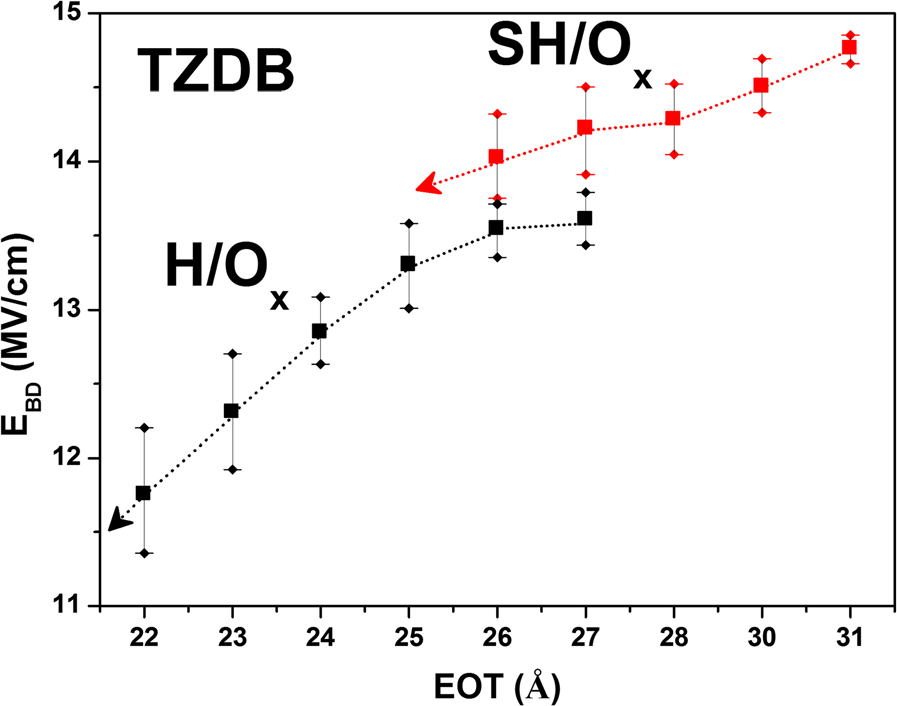
***E***_**BD **_**versus EOT for SH/O**_**x **_**and H/O**_**x**_**.** The *E*_BD_ degraded with thinner IL.

In corroborating that stacking structure owns the higher breakdown field than the one without stacking structure, devices of SH/O_x_ and H/O_x_ were fabricated. Since the platinum was tilted while forming the IL with different thicknesses by ANO, as schematically illustrated in Figure 1, devices with different EOTs were obtained. The C-V curves of SH/O_x_ are shown in Figure 5a, with the overall EOTs ranging from 27 to 22 Ǻ, and the inset shows the corresponding I-V curves. For another sample of H/O_x_, the C-V curves are presented in Figure 5b, with the overall EOTs ranging from 31 to 25 Ǻ, and the I-V curves are presented in the inset. Although both samples have different ranges of EOT, which may result from the longer oxidation time by nitric acid, it does not influence our conclusion since we are comparing the *E*_BD_ instead of breakdown voltage. After the TZDB test, the *E*_BD_ versus different EOTs of SH/O_x_ and H/O_x_ are shown in Figure 6. The result that stacking structure owns larger *E*_BD_ is consistent with our investigation for SH/O and H/O.

**Figure 7:**
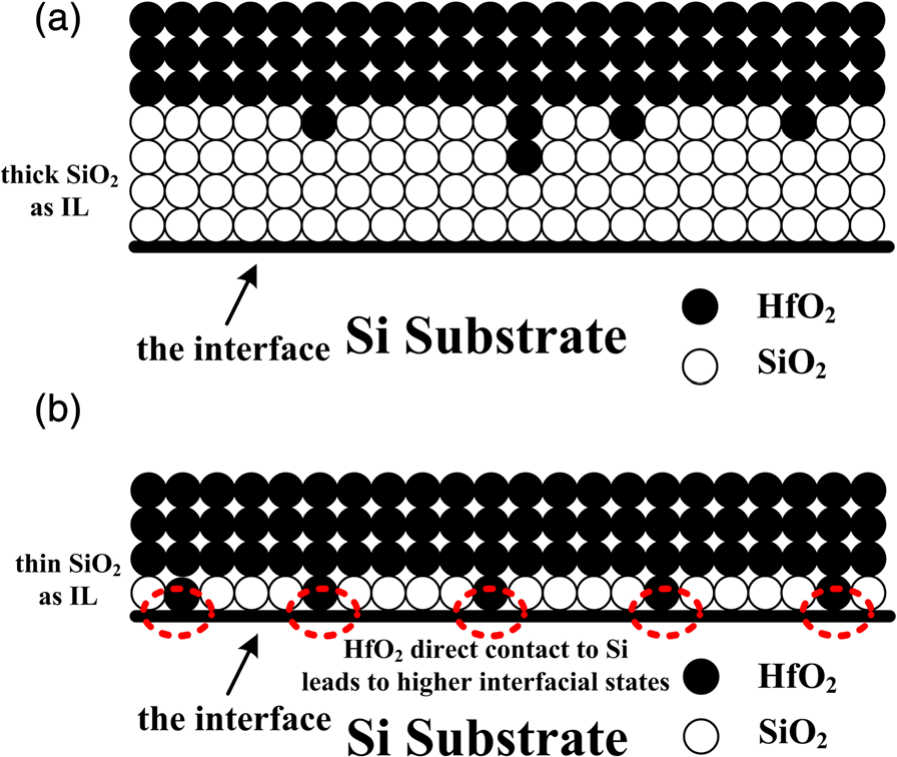
**Structure with thicker and thinner SiO**_**2 **_**as IL. (a)** Structure with thicker SiO_2_ as IL. **(b)** Structure with thinner SiO_2_ as IL.

**Figure 8:**
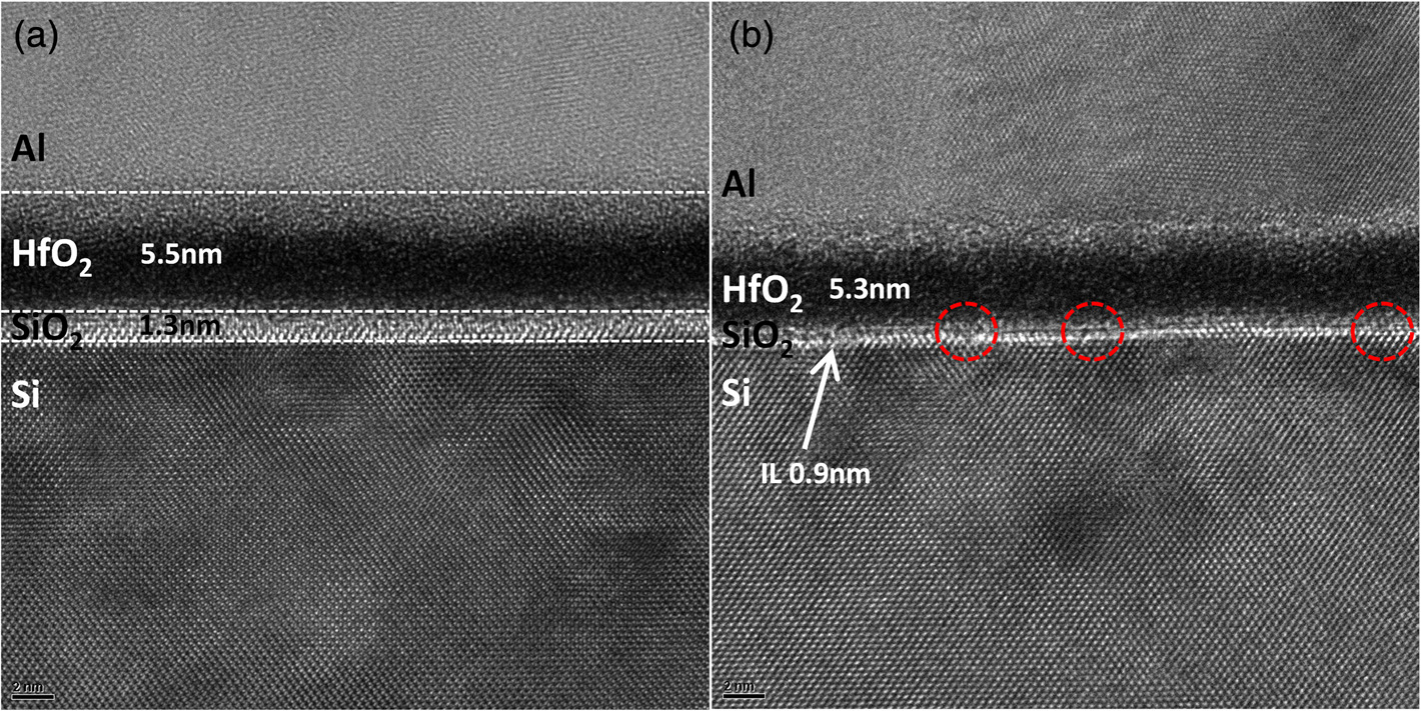
**HRTEM of H/O**_**x **_**with (a) the thickest IL and (b) the thinnest IL.** In **(b)**, it is observed that HfO_2_ is directly contact with Si in some locations.

**Figure 9:**
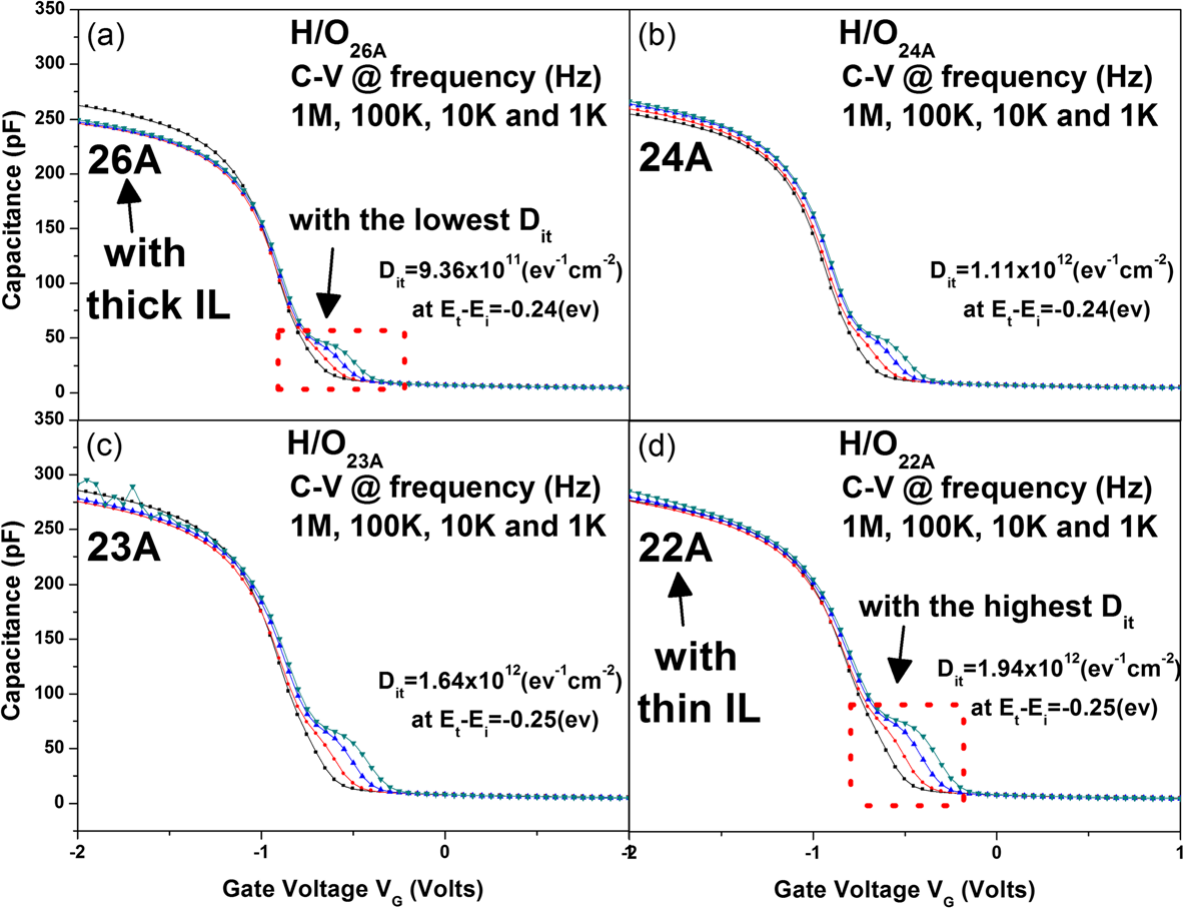
**C-V curves measured at various frequencies for H/O**_**x**_**. (a)** EOT = 26 Å, having the least *D*_it_; **(b)** EOT = 24 Å; **(c)** EOT = 23 Å; **(d)** EOT = 22 Å, having the highest *D*_it_.

**Figure 10:**
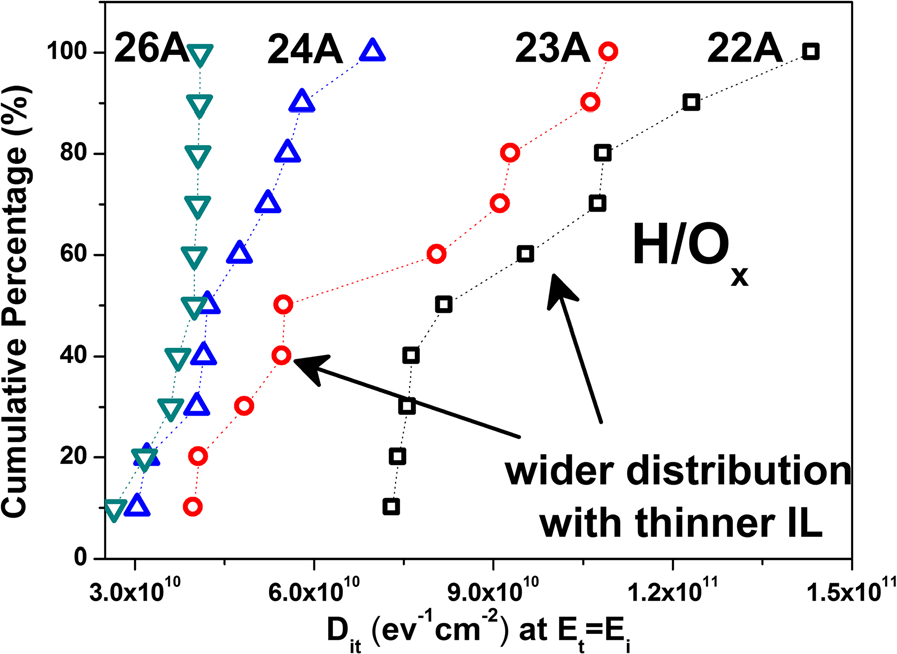
**Cumulative data of *D***_**it **_**at midgap (*E***_***t***_ **=** ***E***_***i***_**) for H/O**_**x**_**.** The wider distribution of data represents the phenomenon of nonuniformity for devices with thinner IL.

Interestingly, it is noticed that through the minimization of EOT in both samples, the *E*_BD_ would all be deteriorated. It is believed that the thin IL is responsible for the phenomenon. SiO_2_ as IL is helpful in relieving the strain due to different lattice constants between high-*κ* dielectric and Si. Furthermore, it helps to reduce the thermodynamic instability between high-*κ* materials and Si. Once the IL becomes thinner, much more HfO_2_ may contact directly to Si, as schematically illustrated in Figure 7a,b for thicker and thinner SiO_2_, respectively. It is believed that thin IL would lead to higher density of interfacial states. The results of HRTEM for H/O_x_ with the thickest and thinnest IL are shown in Figure 8a,b, respectively. The phenomenon that HfO_2_ may directly contact to Si is observed for sample with thin IL, as presented in Figure 8b (red circles). It is consistent with our assumption as described in Figure 7b. Figure 9a,b,c,d shows the C-V curves measured at various frequencies for H/O_x_ with various EOTs (SH/O_x_ not shown for brevity). It is observed that the interface trap density (*D*_it_) is increasing with the decreasing IL thickness. The *D*_it_ could be calculated by using high-low frequency method

(3) $$\begin{array}{l} D_{\mathrm{it}}=\frac{C_{\mathrm{it}}}{q}=\frac{{\left(\frac{1}{C_{L}}-\frac{1}{C_{\mathrm{ox}}}\right)}^{-1}-{\left(\frac{1}{C_{H}}-\frac{1}{C_{\mathrm{ox}}}\right)}^{-1}}{q} \end{array}$$

where *C*_ox_ is the gate oxide capacitance per unit area, *C*_*H*_ is the measured capacitance per unit area under frequency 1 MHz, and *C*_*L*_ is the measured capacitance per unit area under frequency 1 kHz. The cumulative data of *D*_it_ at midgap (*E*_*t*_ = *E*_*i*_) of samples H/O_x_ are presented in Figure 10 (SH/O_x_ not shown for brevity). Higher *D*_it_ and wider Weibull distribution for samples with thin IL are observed. Nonuniform interfacial property becomes serious when IL thickness is reduced.

## Conclusions

In this study, we demonstrated that structure with stacking dielectric layer would own the higher breakdown field from TZDB test. While higher breakdown power at the initiation of breakdown and lower resistance after breakdown are observed for stacking structure. In addition, the importance of IL is discussed in this work. Thinner IL would result in the increase of *D*_it_ and the degradation of breakdown field. The explanation of the phenomenon is proposed and is confirmed by HRTEM.

## Abbreviations

ANO: Anodization; C-V: Capacitance-voltage; EOT: Effective oxide thickness; IL: Interfacial layer; I-V: Current–voltage; NAO: Nitric acid oxidation; TZDB: Time-zero dielectric breakdown.

## Competing interests

The authors declare that they have no competing interests.

## Authors’ contributions

C-SP carried out the experiments and the measurements. J-GH provided thoughts and revised the manuscript. C-SP completed the manuscript. Both authors discussed the results. Both authors read and approved the final manuscript.
